# Supplementary material for: The impact of global selection on local adaptation and reproductive isolation
Source: Philos Trans R Soc Lond B Biol Sci. 2020 Jul 13;375(1806):20190531. doi: 10.1098/rstb.2019.0531 (PMC7423272; doi:10.1098/rstb.2019.0531)
Supplement: Supplementary material [file rstb20190531supp1.pdf]

The impact of global selection on local adaptation and reproductive isolation: supplementary information

Contribution to themed issue of Philosophical Transactions B by Gertjan Bisschop, Derek Setter, Marina Rafajlović, Stuart J.E. Baird and Konrad Lohse

Additional figures trait-based model

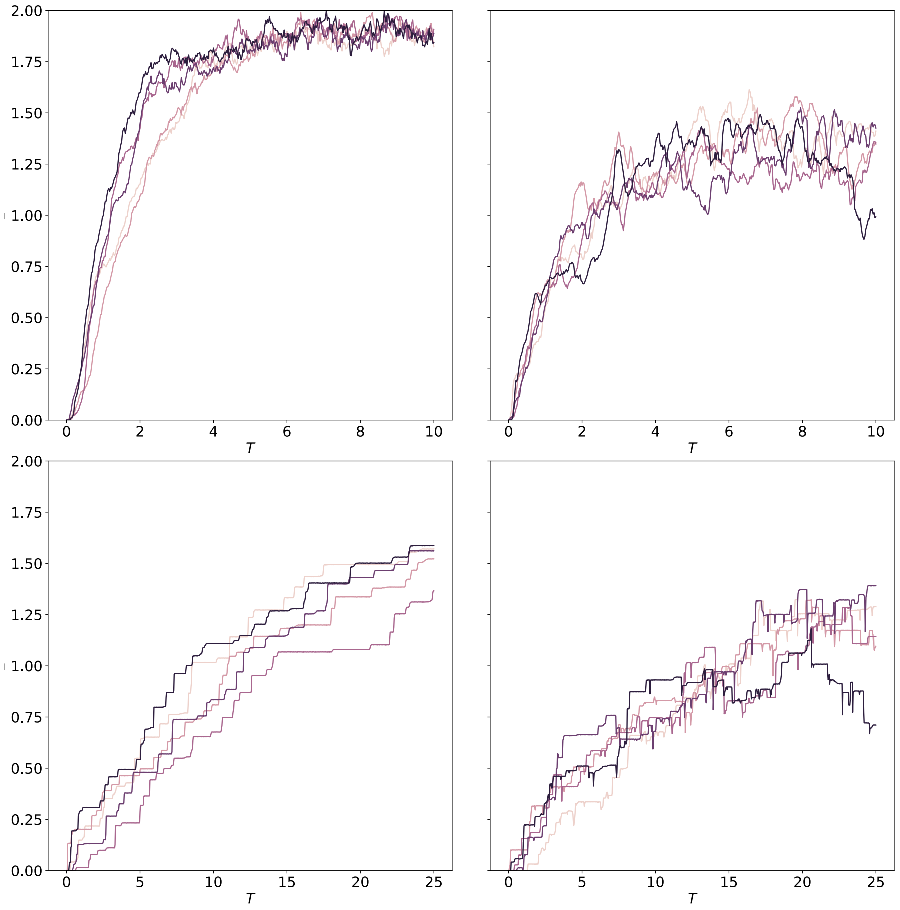

Figure S1: Left: LBMs only, Right: with GBM. Top row: "weak-frequent". Bottom row: "strong-rare". Mean phenotype for the population adapting to θ_+, six replicates are shown for each scenario.

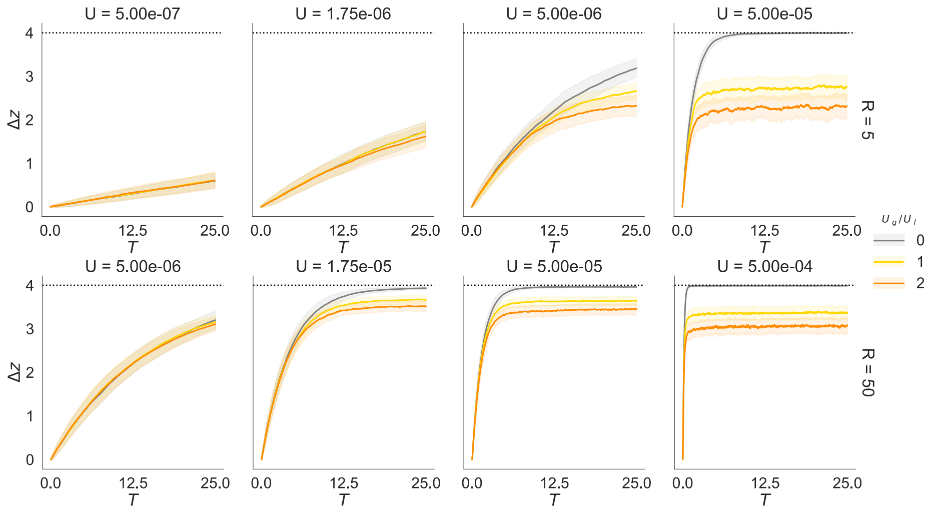

Figure S2: Mean trait divergence (Δz) across 200 replicates (maximum $\Delta z = 4$) for the "strong-rare" case without GBMs (gray) and with GBMs ($U_g/U_l \in \{1,2\}$, coloured lines). The envelopes show 2 standard deviations across replicates (Top $R = 5cM$, bottom $R = 50cM$). We have adapted the mutation rate assuming that the mutational input for an organism with the same average mutation and recombination rate is smaller for a smaller chromosome fragment ($R = 50cM$ vs $R = 5cM$).

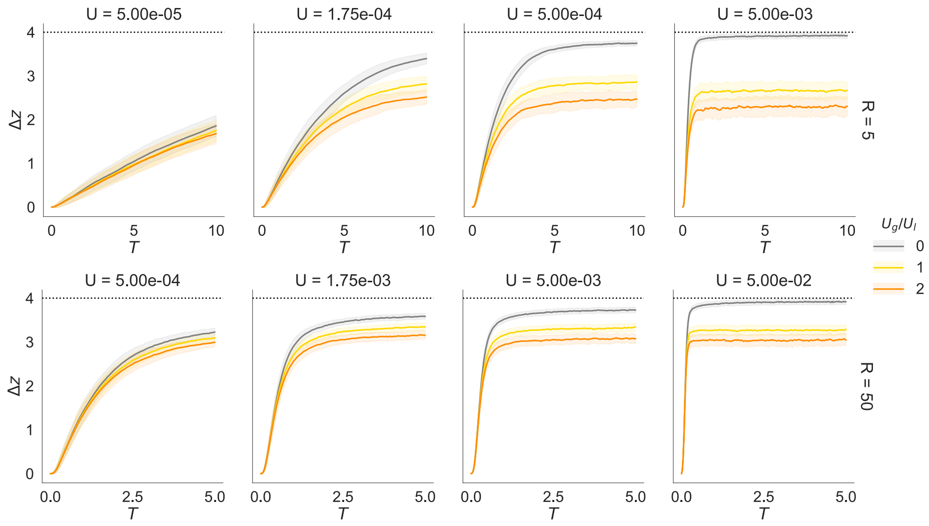

Figure S3: Mean trait divergence (Δz) across 200 replicates (maximum $\Delta z = 4$) for the "weak-frequent" case without GBMs (gray) and with GBMs ($U_g/U_l \in \{1,2\}$, coloured lines). The envelopes show 2 standard deviations across replicates (Top $R = 5cM$, bottom $R = 50cM$). We have adapted the mutation rate assuming that the mutational input for an organism with the same average mutation and recombination rate is smaller for a smaller chromosome fragment ($R\!=\!50cM$ vs $R\!=\!5cM$).

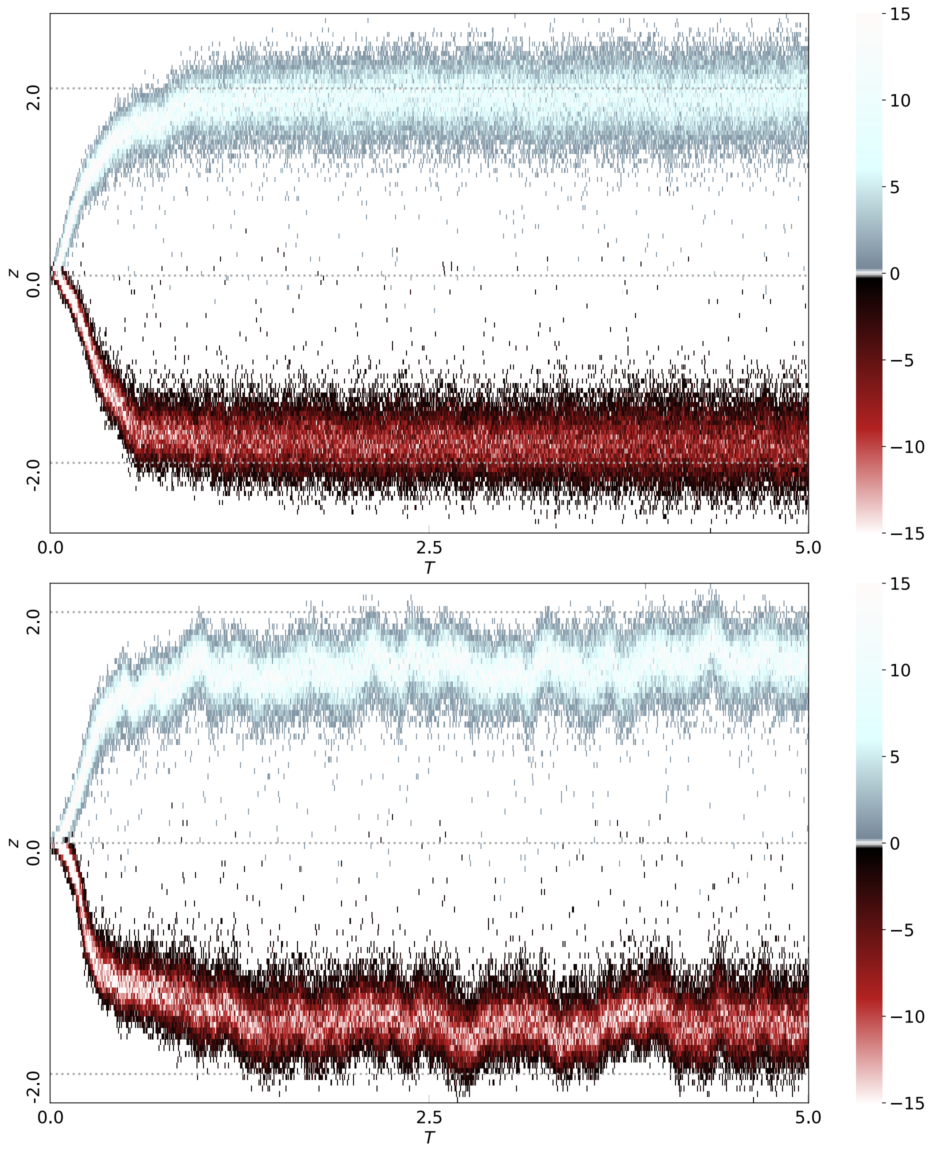

Figure S4: The evolution of individual trait values z for a single run for $5 \times 2N_e$ generations. The local optima are represented by the dotted line ($\theta_- = -2$ and $\theta_+ = 2$). Without (top panel) and with GBMs ($U_g/U_l = 2$, bottom panel) for the "weak-frequent" scenario with $U = 5 \times 10^{-3}$. Individuals (sample of 100 per population) are binned by phenotype. The shade of each dot represents the number of individuals per bin (see colorbar). Note that the higher mutation rate combined with weak selection induces more variance in the population than in the 'strong-rare' scenario.

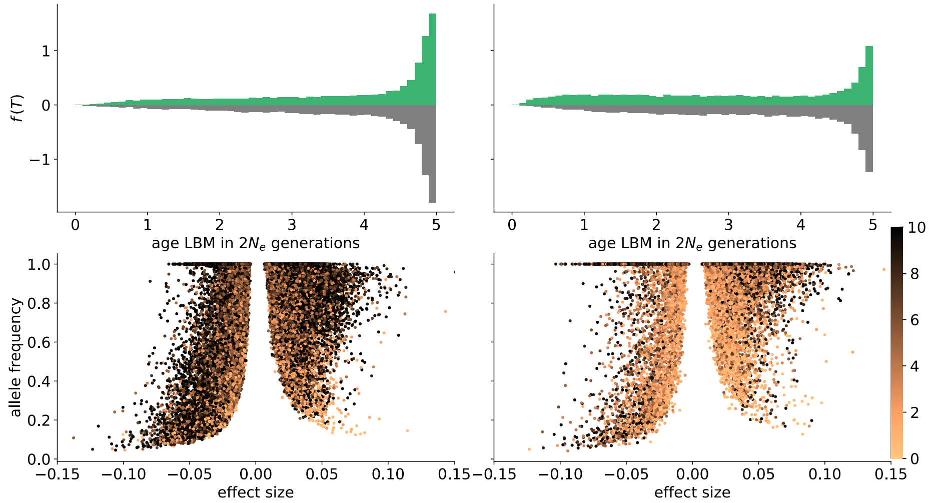

Figure S5: The impact of GBMs on the age distribution of LBMs. Results without GBMs (left) and with GBMs ($U_g/U_l = 2$, right) for "the weak-frequent" scenario, for the population adapting to θ_+, at the dynamic equilibrium phase ($5 \times 2N_e$ generations). Top row: the age distribution of LBMs (across the genome and across 200 replicate runs) weighted by their frequency and effect size. Age is measured in $2N_e$ generation with 0 representing the time of sampling. The histograms on the top (green) and bottom (grey) correspond to LBMs with positive and negative local effects respectively. Bottom row: LBM effect size plotted against the allele frequency. Young and old LBMs grade from orange to black respectively (see color bar).

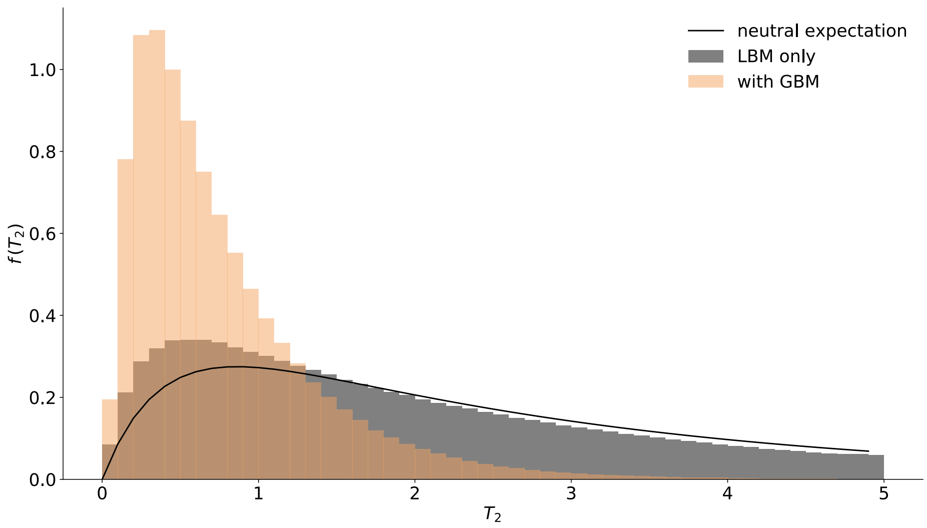

Figure S6: The genome-wide distribution of between population coalescence times $f(T_2)$ for the "weak-frequent" scenario. The neutral expectation (assuming $M = 1$) (**?**, eq. 10) is shown as a gray solid line, distributions. The absence and presence of GBMs ($U_g/U_l = 2$) are shown in gray and orange respectively. 87% and 99% of coalescence times respectively are smaller then $5N_e$. This data corresponds to figure S3 panel (2,3). X-axis is truncated at $5N_e$.

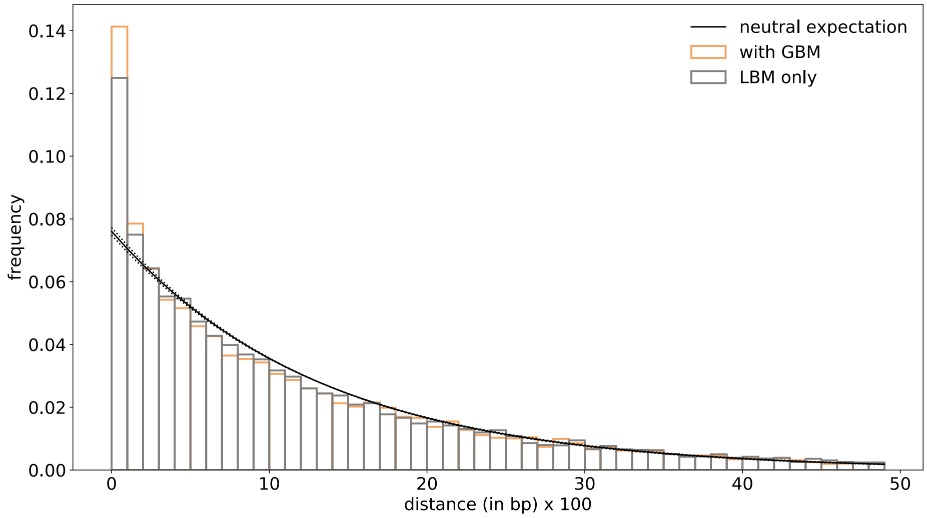

Figure S7: The distribution of pairwise distances between consecutive LBMs contributing positively to divergence (the 75 LBMs of largest effect) for the "weak-frequent" case after $5N_e$ generations. Frequencies are weighted by the mean contribution of each pair of LBM to local adaptation. The black line shows the expected exponential distribution of pairwise distances in the absence of clustering, i.e. assuming loci are distributed uniformly at random (dotted line is 95% confidence interval).

Additional figures sweep-based model

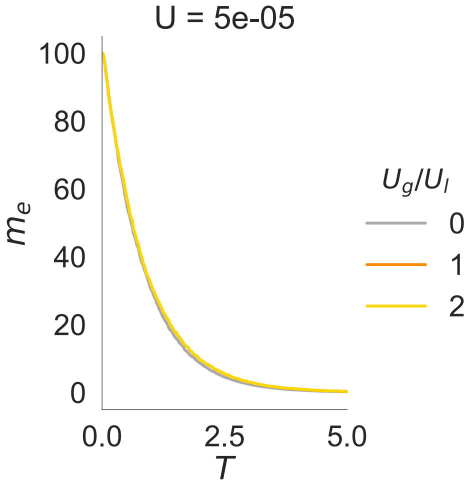

Figure S8: The relative fitness of migrants for the "strong-rare" scenario in the sweep-based model (BU2S) (**?**). Time is measured in $2N_e$ generations. $U_g/U_l \in \{0,1,2\}$, gray, yellow and orange respectively.

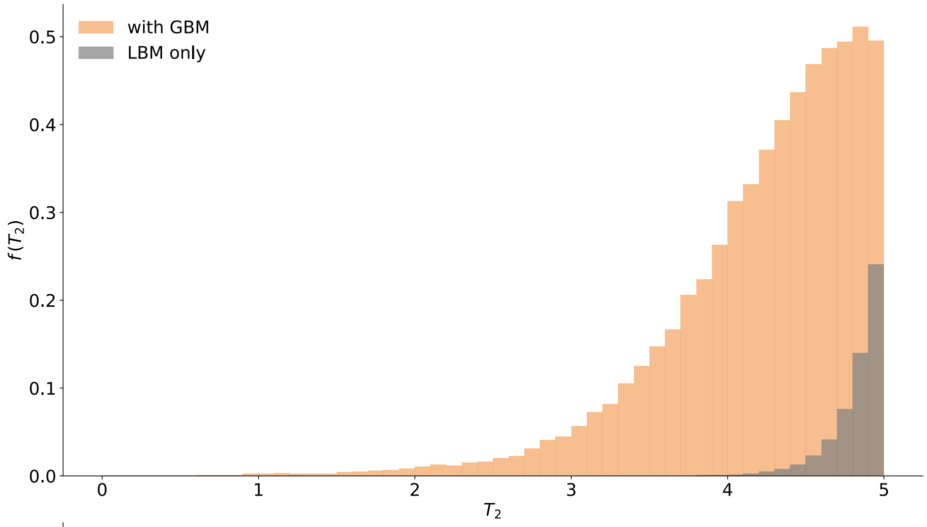

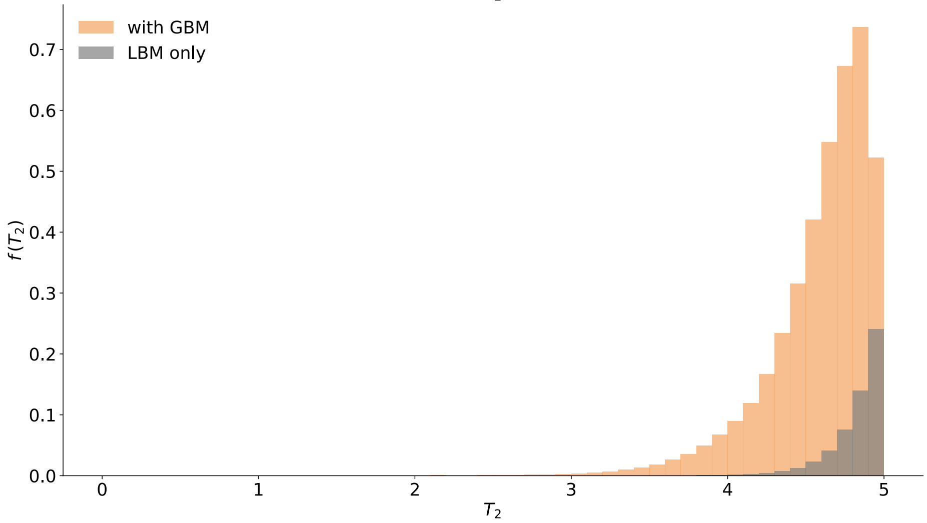

Figure S9: The genome-wide distribution of pairwise coalescence times $f(T_2)$ for the sweep-based model (top: ”strong-rare”, bottom: ”weak-frequent”). Time in $2N_e$ generations, $U_g/U_l = 2$. 0.05% (LBMs only) and 60% (with GBMs) of coalescence times are smaller then 5 (”strong-rare”) and 0.05% (LBMs only) and 40% (with GBMs) for the ”weak-frequent” case.
